# Validation of T-Track® CMV to assess the functionality of cytomegalovirus-reactive cell-mediated immunity in hemodialysis patients

**DOI:** 10.1186/s12865-017-0194-z

**Published:** 2017-03-07

**Authors:** Bernhard Banas, Carsten A. Böger, Gerhard Lückhoff, Bernd Krüger, Sascha Barabas, Julia Batzilla, Mathias Schemmerer, Josef Köstler, Hanna Bendfeldt, Anne Rascle, Ralf Wagner, Ludwig Deml, Joachim Leicht, Bernhard K. Krämer

**Affiliations:** 1 Department of Nephrology, University Medical Center Regensburg, Regensburg, Germany; 2 Dialysis Center Landshut, Landshut, Germany; 3 5th Department of Medicine, University Medical Center Mannheim, Medical Faculty Mannheim of the University Heidelberg, Mannheim, Germany; 4 Lophius Biosciences GmbH, Regensburg, Germany; 5 Institute of Clinical Microbiology and Hygiene, University of Regensburg, Regensburg, Germany; 6 Dialysis Center Schwandorf, Schwandorf, Germany

**Keywords:** Cytomegalovirus, CMV, IE-1, pp65, Cell-mediated immunity, IFN-γ ELISpot, T-Track® CMV, QuantiFERON®-CMV, iTAg™ MHC Tetramers, Hemodialysis

## Abstract

**Background:**

Uncontrolled cytomegalovirus (CMV) replication in immunocompromised solid-organ transplant recipients is a clinically relevant issue and an indication of impaired CMV-specific cell-mediated immunity (CMI). Primary aim of this study was to assess the suitability of the immune monitoring tool T-Track® CMV to determine CMV-reactive CMI in a cohort of hemodialysis patients representative of patients eligible for renal transplantation. Positive and negative agreement of T-Track® CMV with CMV serology was examined in 124 hemodialysis patients, of whom 67 (54%) revealed a positive CMV serostatus. Secondary aim of the study was to evaluate T-Track® CMV performance against two unrelated CMV-specific CMI monitoring assays, QuantiFERON®-CMV and a cocktail of six class I iTAg™ MHC Tetramers.

**Results:**

Positive T-Track® CMV results were obtained in 90% (60/67) of CMV-seropositive hemodialysis patients. In comparison, 73% (45/62) and 77% (40/52) positive agreement with CMV serology was achieved using QuantiFERON®-CMV and iTAg™ MHC Tetramer. Positive T-Track® CMV responses in CMV-seropositive patients were dominated by pp65-reactive cells (58/67 [87%]), while IE-1-responsive cells contributed to an improved (87% to 90%) positive agreement of T-Track® CMV with CMV serology. Interestingly, T-Track® CMV, QuantiFERON®-CMV and iTAg™ MHC Tetramers showed 79% (45/57), 87% (48/55) and 93% (42/45) negative agreement with serology, respectively, and a strong inter-assay variability. Notably, T-Track® CMV was able to detect IE-1-reactive cells in blood samples of patients with a negative CMV serology, suggesting either a previous exposure to CMV that yielded a cellular but no humoral immune response, or TCR cross-reactivity with foreign antigens, both suggesting a possible protective immunity against CMV in these patients.

**Conclusion:**

T-Track® CMV is a highly sensitive assay, enabling the functional assessment of CMV-responsive cells in hemodialysis patients prior to renal transplantation. T-Track® CMV thus represents a valuable immune monitoring tool to identify candidate transplant recipients potentially at increased risk for CMV-related clinical complications.

**Electronic supplementary material:**

The online version of this article (doi:10.1186/s12865-017-0194-z) contains supplementary material, which is available to authorized users.

## Background

Cytomegalovirus (CMV) is a major cause of infectious complications in immunocompromised individuals. Protection against CMV infection or reactivation is normally assured by both the innate and adaptive arms of the immune system [1, 2]. While the humoral and innate responses are essential for the early response to infection [1, 3, 4], cellular immunity is required to control latency and prevent CMV reactivation in latently infected individuals [1]. CD8^+^ cytotoxic T cells (CTL) and CD4^+^ T helper (Th) cells are both required to assure efficient immune protection against CMV reactivation [1, 5–8]. Primary infection is dominated by CD8^+^ T cell response, preferentially targeting CMV immediate early-1 (IE-1) antigen, while long-term recovery is dominated by CD4^+^ T cell response and a switch of reactivity toward CMV lower matrix phosphoprotein 65 (pp65) [6, 8–11]. The frequency of CMV-specific CD8^+^ and CD4^+^ T cells is highly variable, both between healthy CMV-seropositive individuals and during the course of CMV reactivation, and correlates with varying levels of protection [6, 9, 11–13]. Beside changes in T cell frequency, alterations in T cell functionality are associated with impaired response to chronic viral infection [14–17].

Functional impairment of cell-mediated immunity (CMI) in the course of immunosuppression, such as in solid-organ transplant recipients, is a major cause of uncontrolled CMV replication and related clinical complications [18–21]. Treatment regimens with antivirals are costly and associated with harmful side effects. Assessment of CMV-specific immunity may be beneficial to identify patients at increased risk of viral complications, possibly allowing personalized adjustment of antiviral and immunosuppressive therapies.

Various experimental approaches exist to measure CMV-specific CMI. Direct T cell staining with for instance class I iTAg™ MHC Tetramers (Beckman Coulter) allows the quantification of epitope-specific CD8^+^ cells by flow cytometry [18, 22–24]. The sensitivity of tetramer-based assays strictly depends on the coverage of the patient population by the selected HLA types, and this method cannot assess the functionality of CD8^+^ cells. Several assays assessing CMV-specific T cell function have been described. Principally, they measure the production of activation markers (e.g. cytokines such as IFN-γ) in response to antigen stimulation, using intracellular cytokine staining followed by flow cytometry [6, 7, 12, 13, 25, 26], ELISA [21, 27–30], or ELISpot [31–33] assays. These approaches differ not only in their read-out format but also in the antigen (peptide vs. protein) used for the *ex vivo* stimulation. Peptide-based immune monitoring tests such as QuantiFERON®-CMV (Qiagen) allow the quantification of IFN-γ produced by epitope-specific CD8^+^ T cells. Whole blood samples are stimulated with a pool of 22 immunogenic peptides (mapping at IE-1, IE-2, pp28, pp50, pp65 and gB CMV antigens) and covering > 98% of HLA class-I haplotypes. Reactive CD8^+^ T cells are monitored by quantifying secreted IFN-γ by ELISA [34]. QuantiFERON®-CMV was used in a number of studies to assess the risk of CMV reactivation and related disease following solid-organ transplantation [21, 27–30]. A disadvantage of QuantiFERON®-CMV is that it does not assess CMV-specific CD4^+^ T cell function and that it often yields indeterminate results that cannot be interpreted [28, 35, 36]. T-Track**®** CMV is based on the stimulation of freshly isolated peripheral blood mononuclear cells (PBMC) with recombinant urea-formulated (T-activated®) immunodominant CMV IE-1 and pp65 proteins, and the subsequent quantification of antigen-reactive effector cells using an IFN-γ ELISpot assay. T-activated® proteins (*a*proteins) are processed via the exogenous and endogenous antigen processing pathways, resulting in the presentation of naturally-generated peptides by MHC class I and class II molecules, thus enabling the stimulation of a broad spectrum of CMV-protective cells including CD8^+^ and CD4^+^ T cells, as well as the bystander activation of NK and NKT-like cells [37, 38]. As such, T-Track**®** CMV is not restricted to particular HLA types. Performance of T-Track**®** CMV has been recently characterized [38]. A recent study demonstrated its high sensitivity in evaluating changes in CMV-specific CMI during and after pregnancy [39].

Primary aim of this cross-sectional prospective multicenter study was to evaluate the suitability of T-Track® CMV to assess the functionality of CMV-specific CMI in a cohort of patients on hemodialysis due to end-stage renal failure, and thus being representative of patients prior to renal transplantation. Secondary aim of the study was to compare the performance of T-Track® CMV to that of QuantiFERON®-CMV and iTAg™ MHC Tetramers in terms of positive and negative agreement with CMV serology (gold standard reference).

## Methods

### Study design and participants

Hemodialysis patients of any gender and race aged at least 18 years were recruited in the study. Patients requiring systemic immunosuppressive treatment within the last 3 months before study inclusion or suffering from chronic or uncontrolled infections (e.g. HIV or chronic hepatitis) were ineligible for study participation. All subjects gave written informed consent. The study was registered and approved according to the rules, at the German Institute of Medical Documentation and Information (DIMDI). Patient enrolment was started only after receiving the exemption of the permit requirement by the BfArM (Federal Institute for Drugs and Medical Devices) and approval by the ethics committee of the University of Regensburg (approval number 11-122-0205). For reasons of transparency and completeness, the study was prospectively registered at clinicaltrials.gov. The authors confirm that all ongoing and related trials for this intervention are registered at clinicaltrials.gov.

### Blood collection

Lithium heparinized whole blood was collected during routine withdrawal, prior to the start of the dialysis session. For T-Track® CMV and iTAg™ MHC Tetramer staining, 15 ml blood was collected for further PBMC isolation. For QuantiFERON®-CMV, 0.8 to 1.2 ml whole blood was collected into each of the three assay tubes. CMV serology was performed from 2.6 ml whole blood.

### CMV serology

Anti-CMV serological testing was performed using fully automated anti-CMV IgM and IgG tests on the BEP III system (Siemens Healthcare, Eschborn, Germany). CMV IgG-serology was used as primary reference measurement procedure (gold standard method).

### CMV-specific cellular immunity assays

T-Track® CMV (Lophius Biosciences GmbH, Regensburg, Germany) was performed according to manufacturer’s instructions. Briefly, PBMC were isolated and stimulated individually with T-activated® CMV-specific immediate-early 1 (*a*IE-1) and phosphoprotein pp65 (*a*pp65) proteins for 19 h at 37°C. Staphylococcus enterotoxin B (SEB) and medium served as positive and negative controls for the stimulation, respectively. IFN-γ ELISpot assays were performed following manufacturer’s recommendations. IFN-γ-specific spot-forming cells (SFC) were enumerated on a Bioreader® 5000 Pro-Eα (BIO-SYS GmbH, Karben, Germany). Test results were considered positive if the geometric mean of the spots resulting from at least one of the *a*pp65 and *a*IE1 stimulations was ≥ 10 SFC/200,000 PBMC and when the ratio of the geometric means of stimulated and non-stimulated conditions was ≥ 2.5. Positivity rules were calculated as described in the Statistics section.

The QuantiFERON®-CMV assay (Qiagen, Hilden, Germany) was performed according to manufacturer’s instructions. Briefly, QuantiFERON®-CMV collection tubes (Nil Control, CMV Antigen and Mitogen Control) were incubated for 16–24 h at 37°C. IFN-γ levels were determined by enzyme-linked immunosorbent assay (ELISA). Calculation of results was achieved using QuantiFERON®-CMV Analysis Software. QuantiFERON®-CMV test results were considered positive when IFN-γ level (IU/mL) in the CMV Antigen-specific assay minus that in the Nil Control was ≥ 0.2, as recommended by the manufacturer. ELISA measurements are accurate up to 10 IU/mL. Values ≥ 10 IU/mL cannot be quantitatively evaluated.

For the CMV-specific tetramer assay, CMV peptide-specific CD8^+^ T cells were quantified by flow cytometry using a mixture of six class I iTAg™ MHC Tetramers (Beckman Coulter), including: MHC A*0101 Class I Tetramer CMV pp50 (VTEHDTLLY), MHC A*0201 Class I Tetramer CMV pp65 (NLVPMVATV), MHC A*2402 Class I Tetramer CMV pp65 (QYDPVAALF), MHC B*0702 Class I Tetramer CMV pp65 (TPRVTGGGAM), MHC B*0801 Class I Tetramer CMV IE-1 (ELRRKMMYM) and MHC B*3501 Class I Tetramer CMV pp65 (IPSINVHHY). iTAg™ MHC Negative Tetramer PN T01044 (Beckman Coulter) served as negative control. Preselected HLA types are predicted to cover at least 80% of the Caucasian population [40]. Each 1×10^6^ PBMC were stained with 10 μl tetramer mix, 10 μl anti-CD8-FITC (T8-FITC, Beckman Coulter) and 5 μl human CD3 APC-Alexa Fluor® 750 conjugate (Invitrogen/Thermo Fisher Scientific) for 30 min at room temperature protected from light. Cells were washed once in PBS and incubated 45 min at 4 °C protected from light. Dead cells were further stained with SYTOX® RED dead cell stain (Invitrogen/Thermo Fisher Scientific) for 15 min at 4 °C protected from light, prior to flow cytometry analysis. Measurements were performed using a Cytomics FC 500 MPL cytometer (Beckmann Coulter), gating on living and CD3-positive cells. Cell count from the iTAg™ MHC Tetramer Negative staining was subtracted from that of the iTAg™ MHC Tetramer CMV-specific cocktail staining. Data were expressed as the % of CMV-specific tetramer-positive CD8^+^ T cells relative to total CD8^+^ T cells. Test results were considered positive, when the proportion of tetramer-positive CD8^+^ cells was ≥ 0.1% of total CD8^+^ T cells.

### Statistics

Calculations were performed with SAS 9.2 Software and VFP (Variance Function Program) 10.0. Figures were generated using GraphPad Prism. In case of categorically scaled data, absolute and relative frequencies were reported. For continuously scaled data, mean, median, geometric mean, standard deviation, minimum and maximum have been reported. Diagnostic accuracies (sensitivity and specificity) were analyzed from 2 × 2 contingency tables referring bivariate test results to CMV serostatus (reference method). Since the reference standard was not disease but a comparative method, the terms “percent positive agreement” and “percent negative agreement” were used instead of “sensitivity” and “specificity” respectively. The measures are reported with their exact Pearson-Clopper confidence intervals. Kappa (κ) according to Altman and McNemar’s test were used to indicate overall agreement and consistency of pairs of methods respectively. Significance was accepted at *p* < 0.05.

The cut-off of T-Track® CMV positivity was determined using z-statistics (α-level = 0.05) on log10-transformed geometric mean values. Values = 0 were replaced by values near detection limit, which was assumed to be 0.5. Intra-assay standard deviation (SD) of ELISpot measurements from the hemodialysis patient cohort (*n* = 124) and from a cohort of healthy donors (*n* = 45; [38]) was calculated. SD was for the unstimulated control, IE-1 stimulation and pp65 stimulation 0.199, 0.240 and 0.220 (hemodialysis patients), and 0.234, 0.192 and 0.136 (healthy donors), respectively. Considering an intra-assay SD of 0.2 and assuming that 4 replicates are measured for each negative control and test samples, a criterion that the ratio of geometric means of stimulated to unstimulated values is at least 2.5 was obtained. In addition, precision profiles were generated from both IE-1- and pp65-specific test results, whereby a coefficient of variation (CV) no higher than 40% was used as a limit of acceptance of assay validity to determine the respective limit of quantitation (LoQ). Precision profiles for IE-1- and pp65-specific T-Track® CMV yielded LoQ values of 7.8 and 8.3 respectively (see Additional file 1). Comparable limits of quantitation were obtained from precision profiles generated from T-Track® CMV assays performed on PBMC from healthy donors [38]. Based on these analyses, a technical cut-off of 10 SFC/200,000 PBMC was chosen. Altogether, T-Track® CMV test results were considered positive if the geometric mean of the spots resulting from at least one of *a*pp65 and *a*IE1 stimulations was ≥ 10 SFC/200,000 PBMC and if the ratio of the geometric means of stimulated to non-stimulated conditions was ≥ 2.5.

## Results

### Patient characteristics

One hunderd twenty-four hemodialysis patients (68 men, 56 women, mean age 65 ± 13 years) were enrolled in this study. The mean duration of dialysis was 1,913 days (range 21 to 11,640 days). A positive CMV-IgG serostatus was measured in 67/124 (54%) of hemodialysis patients (Table 1). Blood was collected before the start of the dialysis session. Routine blood parameters and inflammation markers are depicted in Table 1.

**Table 1:** Demographic and blood parameters of hemodialysis patients

Age (years), mean ± SD (range)	65 ± 13 (26; 88)
Gender, N (%)	
Male	68 (54.8%)
Female	56 (45.2%)
CMV serostatus, N (%)	
Positive	67 (54%)
Negative	57 (46%)
Duration of dialysis (days), mean ± SD (range)	1,913 ± 2,079 (21; 11,640)
Blood count, mean ± SD (range)	
Hemoglobin (g/dl)	11.3 ± 1.2 (7.8; 16.1)
Erythrocytes (Tpt/l)	3.7 ± 0.44 (2.5; 5.2)
Leukocytes (pt/nl)	7.5 ± 2.4 (3.0; 17.8)
Thrombocytes (Tsd/μl)	234 ± 65 (84; 426)
Inflammation marker, mean ± SD (range)	
CRP (mg/l)^a^	9.7 ± 17.6 (1.0; 143.0)
Absolute number of PBMC x 10^6^ / 15 ml whole blood (mean ± SD (range)	13.5 ± 9.8 (3.2; 87.3)

^a^CRP values were available for 80 out of 124 patients

One hunderd twenty-four T-Track® CMV, 123 QuantiFERON®-CMV and 97 iTAg™ MHC Tetramer measurements were carried out from whole-blood (QuantiFERON®-CMV) or from freshly isolated PBMC (T-Track® CMV, iTAg™ MHC Tetramers). Insufficient amount of blood and/or PBMC did not allow the performance evaluation of all tests for all 124 patients. CMV serology served as a primary reference measurement procedure (gold standard reference) in the performance study.

### Performance of T-Track® CMV

**Fig. 1:**
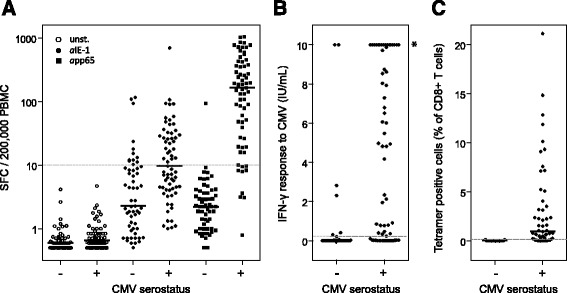
CMV-specific immunity in hemodialysis patients measured with T-Track® CMV (**a**), QuantiFERON®-CMV (**b**) and iTAg™ MHC Tetramers (**c**). **a** Spot-forming cells (SFC) in IFN-γ ELISpot after in vitro stimulation of PBMC from CMV-seronegative (*n* = 57) and CMV-seropositive (*n* = 67) hemodialysis patients with T-activated® *a*IE-1 and *a*pp65 proteins, or with medium (unst.) as a negative control. SFC levels are presented as log10-transformed values in scatter plots, including median values (horizontal *black* lines). The *horizontal grey dashed line* indicates the positivity cut-off (10 SFC / 200,000 PBMC). **b** CD8^+^-secreted IFN-γ levels were measured by ELISA following the stimulation of whole blood from CMV-seronegative (*n* = 57) and CMV-seropositive (*n* = 66) hemodialysis patients with HLA class I-specific peptides. Test results were considered positive when IFN-γ levels ≥ 0.2 IU/mL (*grey dashed line*). Indeterminate results (4/66 seropositive and 2/57 seronegative patients) are not represented; therefore the scatter plots represent the results of 62 seropositive and 55 seronegative assays. *, values ≥ 10 IU/mL cannot be quantitatively evaluated; consequently, no median values were depicted. **c** PBMC of CMV-seronegative (*n* = 45) and CMV-seropositive (*n* = 52) hemodialysis patients were stained with a mixture of six iTAg™ MHC class I Tetramers, and CMV peptide-specific CD8^+^ T cells were quantified by flow cytometry. Test results were considered positive when ≥ 0.1% of total CD8^+^ T cells were tetramer-positive (*grey dashed line*). The scatter plots show median values (*horizontal black lines)*

Positive and negative agreement of T-Track® CMV with CMV serology was investigated using PBMC samples from 124 hemodialysis patients. 58 of the 67 CMV-seropositive patients showed a positive response to *a*pp65 (Table 2) with a median of 165 spot-forming cells (SFC)/200,000 PBMC and a maximum of 1,040 SFC/200,000 PBMC (Fig. 1a). 33 of the 67 CMV-seropositive patients demonstrated an *a*IE-1-specific response in the T-Track® CMV ELISpot assay, with a median of 9.7 SFC/200,000 PBMC and a maximum of 696 SFC/200,000 PBMC (Table 2 and Fig. 1a). By taking into account the outcome of both *a*IE-1 and *a*pp65 stimulations, T-Track® CMV results were positive in 60 out of 67 CMV-seropositive patients, corresponding to an overall positive agreement with CMV serology of 89.6% (Table 2).

**Table 2:** Positive agreement of T-Track® CMV, QuantiFERON®-CMV and iTAg™ MHC Tetramers with CMV serology in hemodialysis patients

Test	CMV positive serology^a^	CMI+	CMI-	Positive agreement	95% CI
T-Track® CMV	67	60	7	0.896	0.797–0.957
CMV *a*IE-1	67	33	34	0.493	0.368–0.618
CMV *a*pp65	67	58	9	0.866	0.760–0.937
QuantiFERON®-CMV^b^	62	45	17	0.726	0.598–0.831
iTAg™ MHC Tetramers	52	40	12	0.769	0.632–0.875

^a^CMV-serology served as primary reference measurement procedure; ^b^calculation of the positive agreement and associated 95% CI do not take into account the 4 indeterminate QuantiFERON®-CMV results out of the 66 CMV-seropositive patients; *CMI+* positive test result, *CMI-* negative test result, *CI* confidence interval

Interestingly, 12 out of 57 (21.1%) study participants who scored negative in a conventional serological assay presented with CMV-reactive effector cells in T-Track® CMV, equivalent to a negative agreement with CMV serology of 78.9% (Table 3). With one exception, positive T-Track® CMV results in CMV-seronegative patients were observed in *a*IE-1-stimulated PBMC samples, and were mostly associated with low spot counts (10.3–23.6 SFC/200,000 PBMC in 9 out 12 patients; Fig. 1a). Three patients however exhibited higher spot counts (93.6–116.5 SFC/200,000 PBMC). In contrast, one *a*pp65-positive test result with a spot count of 94 SFC/200,000 PBMC was observed among the 57 CMV-seronegative patients (Fig. 1a). Of note, this patient also showed a positive test result for *a*IE-1, with 109.2 SFC/200,000 PBMC (Fig. 1a). These observations suggest that T-Track® CMV might have the capacity to detect CMV-reactive cells resulting from a previous CMV infection that was however not sufficient to yield an antibody response.

**Table 3:** Negative agreement of T-Track® CMV, QuantiFERON®-CMV and iTAg™ MHC Tetramers with CMV serology in hemodialysis patients

Test	CMV negative serology^a^	CMI-	CMI+	negative agreement	95% CI
T-Track® CMV	57	45	12	0.789	0.661–0.886
CMV *a*IE-1	57	45	12	0.789	0.661–0.886
CMV *a*pp65	57	56	1	0.982	0.906–1.000
QuantiFERON®-CMV^b^	55	48	7	0.873	0.755–0.947
iTAg™ MHC Tetramers	45	42	3	0.933	0.817–0.986

^a^CMV-serology served as primary reference measurement procedure; ^b^calculation of the negative agreement and associated 95% CI do not take into account the 2 indeterminate QuantiFERON®-CMV results out of the 57 CMV-seronegative patients; *CMI-* negative test result, *CMI+* positive test result, *CI* confidence interval

### Performance of QuantiFERON®-CMV and iTAg™ MHC Tetramers

The QuantiFERON®-CMV assay was performed on blood samples from 66 CMV-seropositive and 57 CMV-seronegative hemodialysis patients. QuantiFERON®-CMV was positive (reactive) in 45/66, negative (non-reactive) in 17/66 and indeterminate in 4/66 of CMV-seropositive patients. Conversely, 7/57, 48/57 and 2/57 of CMV-seronegative patients showed positive, negative and indeterminate test results, respectively. Indeterminate results were excluded from subsequent analyses, as a repetition of the QuantiFERON®-CMV assay from fresh blood samples was not possible. Thus, the results of the QuantiFERON®-CMV assay revealed a positive and negative agreement with CMV serology of 72.6% (45/62) and 87.3% (48/55) respectively (Tables 2 and 3; Fig. 1b).

A mixture of six preselected CMV-specific class I iTAg™ MHC Tetramers based on IE-1, pp65 and pp50 epitopes and predicted to cover at least 80% of the Caucasian population was used to quantify the proportion of CMV-specific CTL in freshly isolated PBMC of 52 CMV-seropositive and 45 CMV-seronegative hemodialysis patients. In these experiments, 40/52 (76.9%) of CMV-seropositive patients were test-positive with a median proportion of 0.98% CMV-specific CD8^+^ T cells / total CD8^+^ T cells and a maximum of 21.1% tetramer-positive CD8^+^ T cells (Table 2 and Fig. 1c). Among 45 CMV-seronegative patients 3 were assay-positive, corresponding to a negative agreement with CMV-serology of 93.3% (Table 3 and Fig. 1c).

### Assessment of agreement between the different assays

The results of T-Track® CMV, QuantiFERON®-CMV and iTAg™ MHC Tetramers were compared to assess their level of agreement. Results of T-Track® CMV moderately agreed with that of QuantiFERON®-CMV (κ = 0.445) and of the CMV iTAg™ MHC Tetramers (κ = 0.434) (Table 4). Statistical analysis of the number of discordant results between T-Track® CMV, QuantiFERON®-CMV and iTAg™ MHC Tetramers using the McNemar’s test revealed that the consistency in the pairs of methods was statistically different between T-Track® CMV and both QuantiFERON®-CMV (*p* = 0.0090) and iTAg™ MHC Tetramers (*p* = 0.0082) (Table 4).

**Table 4:** Assessment of strength (κ) and consistency (McNemar’s Test) of agreement of T-Track® CMV results with QuantiFERON®-CMV and iTAg™ MHC Tetramers results

Test 1	Test 2	κ	95% CI	McNemar
T-Track® CMV	QuantiFERON®-CMV	0.445	0.289–0.601	0.009
T-Track® CMV	iTAg™ MHC Tetramers	0.434	0.264–0.604	0.008

According to Altman, kappa (κ) values between 0.4 and 0.6 refer to moderate agreement. Consistency was evaluated by comparing the number of discordant results using the McNemar’s Test (*p* < 0.05 was considered statistically significant). Of note, assessment does not take into consideration indeterminate results of the QuantiFERON®-CMV assay. *CI* confidence interval

Notably, 14/17 and 12/12 CMV-seropositive patients with negative results in QuantiFERON®-CMV and iTAg™ MHC Tetramer respectively, were assay-positive in T-Track® CMV. Moreover, 5/5 CMV-seropositive patients with negative results for both QuantiFERON®-CMV and iTAg™ MHC Tetramers showed positive T-Track® CMV results. Conversely, the 7 CMV-seropositive patients with a negative T-Track® CMV result showed either positive or negative results by QuantiFERON®-CMV and iTAg™ MHC Tetramers, revealing inter-assay discordance. Interestingly, 3/4 CMV-seropositive patients with indeterminate QuantiFERON®-CMV results showed a positive T-Track® CMV result, of which 2 were also CMV-Tetramer positive. Finally, among the CMV-seronegative patients, only 1 out of 12 positive T-Track® CMV assays was also positive in QuantiFERON®-CMV while 6/7 and 3/3 positive results in QuantiFERON®-CMV and iTAg™ MHC Tetramers respectively were negative in T-Track® CMV.

## Discussion

T-Track® CMV represents a novel assay format, which relies on the functional assessment of various CMV protein-reactive effector cells, including CD4^+^ (Th) cells, CD8^+^ (CTL), NK and NKT-like cells [37, 38], all of which being described to contribute to the clearance of CMV replication [1, 6–8, 41–44]. In this study, the suitability of T-Track® CMV to measure CMV-specific CMI in a cohort of dialysis patients and its performance against QuantiFERON®-CMV [34] and iTAg™ MHC Tetramer assays [24] were evaluated.

T-Track® CMV revealed a positive agreement with CMV-serology of 90% in hemodialysis patients, higher than that measured with QuantiFERON®-CMV (73%) and a mixture of 6 preselected CMV-specific iTAg™ MHC Tetramers (77%), indicating a higher sensitivity of T-Track® CMV compared to QuantiFERON®-CMV and iTAg™ MHC Tetramers. This difference in positive agreement with CMV-serology is likely due to the difference in format of the three assays. Beside the detection of a broad repertoire of CD8^+^ T cells as a result of antigen cross-presentation [37], T-Track® CMV is indeed able to assess the functionality of CMV-reactive CD4^+^ cells but also the bystander activation of IFN-γ-producing NK and NKT-like cells [41, 43–46]. In contrast, QuantiFERON®-CMV and iTAg™ MHC Tetramers are restricted to the detection of selected CMV-specific CD8^+^ cells. In addition to the assay format, the combination of results of the separate measurement of pp65- and IE-1-responsive effector cells by T-Track® CMV contributes to the increased positive agreement with CMV serology, from 87% with pp65-specific CMI alone to 90% with both pp65- and IE-1-specific CMI. This positive contribution of IE-1 to the sensitivity of T-Track® CMV is in agreement with the demonstration that CMV-seropositive healthy donors do not always exhibit a pp65-specific CD8^+^ T cell response and that a non-negligible proportion of individuals only show a CD8^+^ T cell response to IE-1 [47]. Other factors potentially enhancing the sensitivity of T-Track® CMV are the standardization of the assay, which uses a constant number of PBMC (as opposed to whole blood in QuantiFERON®-CMV, possibly resulting in high inter-individual variability), its HLA-type-independence (as opposed especially to the iTAg™ MHC Tetramer assay) and the absence of indeterminate results (as opposed to QuantiFERON®-CMV). In that regard, 4/66 CMV-seropositive and 2/57 CMV-seronegative hemodialysis patients yielded indeterminate results with QuantiFERON®-CMV, which - with a rate of 5% - is lower than what was reported in transplant recipients [28, 35, 36].

Interestingly, the positive agreement of T-Track® CMV with CMV-serology of 90% measured in this cohort of hemodialysis patients was lower than that obtained in CMV-seropositive healthy individuals (97%; [38]). Similarly, the positive agreement of QuantiFERON®-CMV results with CMV-serology in hemodialysis patients (73%) is below the positive agreement of 88% to 97% previously reported in healthy adults [27, 34]. This difference might be explained by a functional impairment of Th cells, CTL, Antigen-presenting cells (APC), NK and NKT cells in patients with end-stage renal failure undergoing hemodialysis [48–52]. A reduced CMV-CMI prior to renal transplantation might be associated with an increased risk of CMV reactivation following transplantation. In support to this proposition, several studies reported an association between impaired CMV-specific CMI pre-transplantation and increased risk for CMV viremia post-transplantation [29, 31, 53]. The high positive agreement of T-Track® CMV with CMV serology observed in this cohort of hemodialysis patients therefore emphasizes the suitability and clinical relevance of T-Track® CMV for patients eligible for renal transplantation.

Although both CMV pp65 and IE-1 antigens contain multiple CD4^+^ and CD8^+^ T cell epitopes presented by different HLA alleles [40], the number of reactive effector cells responding to stimulation with *a*pp65 was substantially higher than that responding to *a*IE-1. This difference might result in part from the dynamics of pp65- and IE-1-reactive CD4^+^ and CD8^+^ T cells in the course of the immune response to CMV infection, long-term seroconversion being dominated by pp65- over IE-1-reactive T cells [8–11, 53, 54]. Moreover, mechanisms of immune evasion involving CMV-encoded unique short (US) proteins and resulting in the inhibition of the MHC-I-dependent antigen presentation pathway appear to be responsible for impaired IE-1 antigen processing and presentation, and thus in the low frequency of IE-1-reactive CD8^+^ T cells [55–57]. On the other hand, differential antigen uptake, processing and presentation by APC, possibly influenced by pp65 and IE-1 intrinsic properties [54, 58, 59], might explain inter-individual differences in the frequency of CMV antigen-specific T cells. Accordingly, comparable CD8^+^ T cell response to IE-1 and pp65 has also been described in some CMV-seropositive healthy donors [47, 60]. The clinical significance of the differential responses to different antigens using T-Track® CMV needs to be elucidated in future studies.

12/57 CMV-seronegative patients revealed positive T-Track® CMV results, corresponding to a negative agreement of T-Track® CMV with CMV serology of 79%. Positive test results were mainly attributed to *a*IE-1 stimulation, and negative agreement raised to 98% when considering the results of *a*pp65 stimulation alone. IE-1-induced spot counts were close to T-Track® CMV positivity threshold in 9/12 patients and only 3 CMV-seronegative patients showed higher IE-1-induced spot counts. We can reasonably rule out false negative CMV serology test results in these patients, as repetition of CMV serology 6 months upon completion of the study in 9 patients who were still available, confirmed their negative serostatus for IgG and IgM (data not shown). Comparatively, 7/55 QuantiFERON®-CMV and 3/45 CMV iTAg™ MHC Tetramer measurements also revealed positive test results in seronegative dialysis patients. Interestingly, with one exception, the 12, 7 and 3 CMV seronegative patients with positive test results in T-Track® CMV, QuantiFERON®-CMV and CMV iTAg™ MHC Tetramers, respectively, were different. This inter-assay variability contributes to the moderate agreement (κ = ~0.4) observed between T-Track® CMV and the 2 alternative assays, and is in agreement with previous studies reporting discordant results between IFN-γ ELISpot and QuantiFERON®-CMV [61, 62]. This inter-assay variability likely reflects differences in the ability of antigen stimulants in each assay to activate distinct subsets of CMV-reactive T cells. Urea formulation of T-activated® CMV antigens increases protein uptake and promotes antigen processing and presentation in the context of both MHC-I (cross-presentation) and MHC-II [37]. T-Track® CMV is thus able to activate a broad range of CD8^+^ and CD4^+^ T cells, encompassing a larger T cell repertoire than QuantiFERON®-CMV or iTAg™ MHC-I Tetramers, and possibly explaining the higher number of CMV-seronegative patients with positive T-Track® CMV test results.

Interestingly, detection of CMV-reactive effector T cells within CMV-seronegative individuals has been described by others, both in healthy individuals and in transplant recipients, at frequencies of 2–11% among healthy donors and up to 30% in renal transplant recipients [13, 63–65]. With 21% CMV-seronegative hemodialysis patients with positive T-Track® CMV results, our data are in concordance with these published studies. Although Sester et al. questioned the accuracy of serologic testing, in particular in case of borderline immunoglobulin titers [65], Loeth et al. elegantly demonstrated in their study on healthy individuals, that the frequency of 11% seronegative donors with pp65-specific CD4^+^ and CD8^+^ response was neither due to wrong serological assignment, nor to immune cross-reactivity with the closely related herpes virus HHV6, nor to *in vitro* priming. Instead, their demonstration that a large proportion of seronegative donors could mount a strong pp65-specific CD4^+^ (and to a lesser extent CD8^+^) response upon *in vitro* stimulation, led the authors to suggest that these individuals were previously exposed to CMV but failed to mount a humoral immune response [63]. We cannot exclude at this point this possibility nor that TCR cross-reactivity with closely related herpes viruses [66, 67] or with environmental antigens [68, 69] is responsible for the detection of CMV-reactive cells in CMV-seronegative hemodialysis patients. On the other hand, we can reasonably exclude the possibility that signals detected by IFN-γ ELISpot following 19 h of antigen stimulation originate from CMV-specific naïve T cells present in the PBMC population [70–72]. Indeed, although antigen-specific naïve T cells can be primed and expanded in vitro and in vivo [69, 72–78], the vast majority of antigen-stimulated naïve T cells produce no IFN-γ and do not divide for the first ~3 days of stimulation [69, 76, 79–82]. The higher proportion of CMV-reactive cells in seronegative dialysis patients in T-Track® CMV compared to QuantiFERON®-CMV and iTAg™ MHC Tetramers supports the hypothesis that IE-1-specific CD4^+^ T cells (and possibly CD8^+^ T cells through cross-presentation) contribute to the detected signals. On the other hand, the increased proportion of IE-1-reactive cells over pp65-reactive cells in CMV-seronegative patients supports the idea of a recent exposure to CMV, as response to primary infection is usually dominated by IE-1-reactive (predominantly CD8^+^) effector cells [6, 8–11]. Additional experiments will be necessary to address these propositions. Whatever the mechanism involved in the generation of CMV-reactive effector cells in CMV-seronegative patients, these observations raise the attractive possibility that these individuals might have a protective immunity against CMV infection. Clearly, further investigations are needed to address this possibility. Altogether, our data suggest that T-Track® CMV exhibits a performance superior to that of QuantiFERON®-CMV and of iTAg™ MHC Tetramers, and possibly also superior to that of CMV-IgG serology, for the detection of possible immunity against CMV.

## Conclusions

T-Track® CMV represents a highly standardized and sensitive assay suitable for the monitoring of CMV-specific cell-mediated immunity in end-stage renal failure patients, representative of patients prior to renal transplantation. Further validation of T-Track® CMV in multi-center clinical studies on kidney and allogeneic stem cell transplant patients is currently on-going, to evaluate its use for the risk assessment and prediction of CMV-related clinical complications in transplant recipients. In these situations, monitoring of CMV-specific CMI could help physicians better define patient populations that would benefit from prophylactic antiviral therapy, and assist the decision as to when to withdraw prophylaxis safely. Reducing prophylactic antiviral treatment would be beneficial in limiting both treatment-related nephrotoxic side effects and costs.

## Additional file

Additional file 1: Precision profiles of the specific response to IE-1 (A) and pp65 (B) in T-Track® CMV. A coefficient of variation (CV) no higher than 40% was used as a limit of acceptance of assay validity to determine the respective limit of quantitation (LoQ). LoQ values determined at CV = 40% for IE-1 (A) and pp65 (B) in the hemodialysis study (*n* = 124) using T-Track® CMV was 7.8 and 8.3 (SFC / 200,000 PBMC) respectively. Comparable LoQ values were obtained from T-Track® CMV assays performed on PBMC from 45 healthy donors [38]. Based on these analyses, a technical cut-off of 10 SFC / 200,000 PBMC was chosen. (PDF 334 kb)

## Abbreviations

APC: Antigen-presenting cell
*a*protein: T-activated® protein
CMI: Cell-mediated immunity
CMV: Cytomegalovirus
CTL: Cytotoxic T lymphocyte
CV: Coefficient of variation
ELISA: Enzyme-linked immunosorbent assay
ELISpot: Enzyme-linked immunospot
HIV: Human immunodeficiency virus
HLA: Human leukocyte antigen
IE-1: Immediate early-1 protein
IFN-γ: Interferon gamma
LoQ: Limit of quantitation
NK: Natural killer cell
NKT: Natural killer T cell
PBMC: Peripheral blood mononuclear cell
pp65: 65 kDa lower matrix phosphoprotein
SD: Standard deviation
SFC: Spot-forming cell
Th: T helper cell

## References

[1] Hanley PJ, Bollard CM (2014). Controlling cytomegalovirus: helping the immune system take the lead. Viruses.

[2] Crough T, Khanna R (2009). Immunobiology of human cytomegalovirus: from bench to bedside. Clin Microbiol Rev.

[3] Schleiss MR (2013). Cytomegalovirus in the neonate: immune correlates of infection and protection. Clin Dev Immunol.

[4] Rossini G, Cerboni C, Santoni A, Landini MP, Landolfo S, Gatti D (2012). Interplay between human cytomegalovirus and intrinsic/innate host responses: a complex bidirectional relationship. Mediators Inflamm.

[5] Jeitziner SM, Walton SM, Torti N, Oxenius A (2013). Adoptive transfer of cytomegalovirus-specific effector CD4+ T cells provides antiviral protection from murine CMV infection. Eur J Immunol.

[6] Sester M, Sester U, Gärtner B, Heine G, Girndt M, Mueller-Lantzsch N (2001). Levels of virus-specific CD4 T cells correlate with cytomegalovirus control and predict virus-induced disease after renal transplantation. Transplantation.

[7] Gamadia LE, Remmerswaal EBM, Weel JF, Bemelman F, van Lier RAW, Ten Berge IJM (2003). Primary immune responses to human CMV: a critical role for IFN-gamma-producing CD4+ T cells in protection against CMV disease. Blood.

[8] Sester M, Sester U, Gärtner BC, Girndt M, Meyerhans A, Köhler H (2002). Dominance of virus-specific CD8 T cells in human primary cytomegalovirus infection. J Am Soc Nephrol JASN.

[9] Widmann T, Sester U, Gärtner BC, Schubert J, Pfreundschuh M, Köhler H (2008). Levels of CMV specific CD4 T cells are dynamic and correlate with CMV viremia after allogeneic stem cell transplantation. PLoS One.

[10] Sacre K, Carcelain G, Cassoux N, Fillet A-M, Costagliola D, Vittecoq D (2005). Repertoire, diversity, and differentiation of specific CD8 T cells are associated with immune protection against human cytomegalovirus disease. J Exp Med.

[11] Sester M, Sester U, Gärtner B, Kubuschok B, Girndt M, Meyerhans A (2002). Sustained high frequencies of specific CD4 T cells restricted to a single persistent virus. J Virol.

[12] Dunn HS, Haney DJ, Ghanekar SA, Stepick-Biek P, Lewis DB, Maecker HT (2002). Dynamics of CD4 and CD8 T Cell Responses to Cytomegalovirus in Healthy Human Donors. J Infect Dis.

[13] Sinclair E, Black D, Epling CL, Carvidi A, Josefowicz SZ, Bredt BM (2004). CMV antigen-specific CD4+ and CD8+ T cell IFNgamma expression and proliferation responses in healthy CMV-seropositive individuals. Viral Immunol.

[14] Sester U, Presser D, Dirks J, Gärtner BC, Köhler H, Sester M (2008). PD-1 expression and IL-2 loss of cytomegalovirus- specific T cells correlates with viremia and reversible functional anergy. Am J Transplant Off J Am Soc Transplant Am Soc Transpl Surg.

[15] Dirks J, Tas H, Schmidt T, Kirsch S, Gärtner BC, Sester U (2013). PD-1 analysis on CD28(-) CD27(-) CD4 T cells allows stimulation-independent assessment of CMV viremic episodes in transplant recipients. Off J Am Soc Transplant Am Soc Transpl Surg.

[16] Engstrand M, Lidehall AK, Totterman TH, Herrman B, Eriksson B-M, Korsgren O (2003). Cellular responses to cytomegalovirus in immunosuppressed patients: circulating CD8+ T cells recognizing CMVpp65 are present but display functional impairment. Clin Exp Immunol.

[17] Huster KM, Stemberger C, Gasteiger G, Kastenmüller W, Drexler I, Busch DH (2009). Cutting edge: memory CD8 T cell compartment grows in size with immunological experience but nevertheless can lose function. J Immunol.

[18] Sund F, Lidehäll A-K, Claesson K, Foss A, Tötterman TH, Korsgren O (2010). CMV-specific T-cell immunity, viral load, and clinical outcome in seropositive renal transplant recipients: a pilot study. Clin Transplant.

[19] Fernández-Ruiz M, Kumar D, Humar A (2014). Clinical immune-monitoring strategies for predicting infection risk in solid organ transplantation. Clin Transl Immunol.

[20] Kotton CN (2010). Management of cytomegalovirus infection in solid organ transplantation. Nat Rev Nephrol.

[21] Lisboa LF, Kumar D, Wilson LE, Humar A (2012). Clinical utility of cytomegalovirus cell-mediated immunity in transplant recipients with cytomegalovirus viremia. Transplantation.

[22] Koehl U, Dirkwinkel E, Koenig M, Erben S, Soerensen J, Bader P (2008). Reconstitution of cytomegalovirus specific T cells after pediatric allogeneic stem cell transplantation: results from a pilot study using a multi-allele CMV tetramer group. Klin Padiatr.

[23] Gratama JW, Boeckh M, Nakamura R, Cornelissen JJ, Brooimans RA, Zaia JA (2010). Immune monitoring with iTAg MHC Tetramers for prediction of recurrent or persistent cytomegalovirus infection or disease in allogeneic hematopoietic stem cell transplant recipients: a prospective multicenter study. Blood.

[24] Klenerman P, Cerundolo V, Dunbar PR (2002). Tracking T cells with tetramers: new tales from new tools. Nat Rev Immunol.

[25] Bunde T, Kirchner A, Hoffmeister B, Habedank D, Hetzer R, Cherepnev G (2005). Protection from cytomegalovirus after transplantation is correlated with immediate early 1-specific CD8 T cells. J Exp Med.

[26] Egli A, Binet I, Binggeli S, Jäger C, Dumoulin A, Schaub S (2008). Cytomegalovirus-specific T-cell responses and viral replication in kidney transplant recipients. J Transl Med.

[27] Abate D, Saldan A, Mengoli C, Fiscon M, Silvestre C, Fallico L (2013). Comparison of cytomegalovirus (CMV) enzyme-linked immunosorbent spot and CMV quantiferon gamma interferon-releasing assays in assessing risk of CMV infection in kidney transplant recipients. J Clin Microbiol.

[28] Manuel O, Husain S, Kumar D, Zayas C, Mawhorter S, Levi ME (2013). Assessment of cytomegalovirus-specific cell-mediated immunity for the prediction of cytomegalovirus disease in high-risk solid-organ transplant recipients: a multicenter cohort study. Clin Infect Dis Off Publ Infect Dis Soc Am.

[29] Cantisán S, Lara R, Montejo M, Redel J, Rodríguez-Benot A, Gutiérrez-Aroca J (2013). Pretransplant interferon-γ secretion by CMV-specific CD8+ T cells informs the risk of CMV replication after transplantation. Am J Transplant Off J Am Soc Transplant Am Soc Transpl Surg.

[30] Kumar D, Chernenko S, Moussa G, Cobos I, Manuel O, Preiksaitis J (2009). Cell-mediated immunity to predict cytomegalovirus disease in high-risk solid organ transplant recipients. Am J Transplant Off J Am Soc Transplant Am Soc Transpl Surg.

[31] Bestard O, Lucia M, Crespo E, Van Liempt B, Palacio D, Melilli E (2013). Pretransplant immediately early-1-specific T cell responses provide protection for CMV infection after kidney transplantation. Am J Transplant Off J Am Soc Transplant Am Soc Transpl Surg.

[32] Godard B, Gazagne A, Gey A, Baptiste M, Vingert B, Pegaz-Fiornet B (2004). Optimization of an elispot assay to detect cytomegalovirus-specific CD8+ T lymphocytes. Hum Immunol.

[33] Schmittel A, Keilholz U, Scheibenbogen C (1997). Evaluation of the interferon-gamma ELISPOT-assay for quantification of peptide specific T lymphocytes from peripheral blood. J Immunol Methods.

[34] Walker S, Fazou C, Crough T, Holdsworth R, Kiely P, Veale M (2007). Ex vivo monitoring of human cytomegalovirus-specific CD8+ T-cell responses using QuantiFERON-CMV. Transpl Infect Dis Off J Transplant Soc.

[35] Clari MÁ, Muñoz-Cobo B, Solano C, Benet I, Costa E, Remigia MJ (2012). Performance of the QuantiFERON-cytomegalovirus (CMV) assay for detection and estimation of the magnitude and functionality of the CMV-specific gamma interferon-producing CD8(+) T-cell response in allogeneic stem cell transplant recipients. Clin Vaccine Immunol CVI.

[36] Fleming T, Dunne J, Crowley B (2010). Ex vivo monitoring of human cytomegalovirus-specific CD8(+) T-Cell responses using the QuantiFERON-CMV assay in allogeneic hematopoietic stem cell transplant recipients attending an Irish hospital. J Med Virol.

[37] Barabas S, Gary R, Bauer T, Lindner J, Lindner P, Weinberger B (2008). Urea-mediated cross-presentation of soluble Epstein-Barr virus BZLF1 protein. PLoS Pathog.

[38] Barabas S, Spindler T, Kiener R, Tonar C, Lugner T, Batzilla J, Bendfeldt H, Rascle A, Asbach B, Wagner R, Deml L (2017). An optimized IFN-γ ELISpot assay for the sensitive and standardized monitoring of CMV protein-reactive effector cells of cell-mediated immunity. BMC Immunol.

[39] Reuschel E, Barabas S, Zeman F, Bendfeldt H, Rascle A, Deml L (2017). Functional impairment of CMV-reactive cellular immunity during pregnancy. J Med Virol.

[40] Bui H-H, Sidney J, Dinh K, Southwood S, Newman MJ, Sette A (2006). Predicting population coverage of T-cell epitope-based diagnostics and vaccines. BMC Bioinformatics.

[41] van Dommelen SLH, Tabarias HA, Smyth MJ, Degli-Esposti MA (2003). Activation of Natural Killer (NK) T Cells during Murine Cytomegalovirus Infection Enhances the Antiviral Response Mediated by NK Cells. J Virol.

[42] Sylwester AW, Mitchell BL, Edgar JB, Taormina C, Pelte C, Ruchti F (2005). Broadly targeted human cytomegalovirus-specific CD4+ and CD8+ T cells dominate the memory compartments of exposed subjects. J Exp Med.

[43] Kamath AT, Sheasby CE, Tough DF (2005). Dendritic cells and NK cells stimulate bystander T cell activation in response to TLR agonists through secretion of IFN-alpha beta and IFN-gamma. J Immunol.

[44] Min-Oo G, Lanier LL (2014). Cytomegalovirus generates long-lived antigen-specific NK cells with diminished bystander activation to heterologous infection. J Exp Med.

[45] Reschner A, Hubert P, Delvenne P, Boniver J, Jacobs N (2008). Innate lymphocyte and dendritic cell cross-talk: a key factor in the regulation of the immune response. Clin Exp Immunol.

[46] Ferlazzo G, Morandi B (2014). Cross-Talks between Natural Killer Cells and Distinct Subsets of Dendritic Cells. Front Immunol.

[47] Kern F, Surel IP, Faulhaber N, Frömmel C, Schneider-Mergener J, Schönemann C (1999). Target structures of the CD8(+)-T-cell response to human cytomegalovirus: the 72-kilodalton major immediate-early protein revisited. J Virol.

[48] Litjens NHR, Huisman M, van den Dorpel M, Betjes MGH (2008). Impaired immune responses and antigen-specific memory CD4+ T cells in hemodialysis patients. J Am Soc Nephrol JASN.

[49] Girndt M, Sester U, Sester M, Kaul H, Köhler H (1999). Impaired cellular immune function in patients with end-stage renal failure. Nephrol Dial Transplant Off Publ Eur Dial Transpl Assoc - Eur Ren Assoc.

[50] Girndt M, Sester M, Sester U, Kaul H, Köhler H (2001). Defective expression of B7-2 (CD86) on monocytes of dialysis patients correlates to the uremia-associated immune defect. Kidney Int.

[51] Lonnemann G (2008). Impaired NK, cell function in ESRD patients. Blood Purif.

[52] Mattes FM, Vargas A, Kopycinski J, Hainsworth EG, Sweny P, Nebbia G (2008). Functional impairment of cytomegalovirus specific CD8 T cells predicts high-level replication after renal transplantation. Am J Transplant Off J Am Soc Transplant Am Soc Transpl Surg.

[53] Gerna G, Lilleri D, Fornara C, Comolli G, Lozza L, Campana C (2006). Monitoring of human cytomegalovirus-specific CD4 and CD8 T-cell immunity in patients receiving solid organ transplantation. Am J Transplant Off J Am Soc Transplant Am Soc Transpl Surg.

[54] Tabi Z, Moutaftsi M, Borysiewicz LK (2001). Human cytomegalovirus pp 65- and immediate early 1 antigen-specific HLA class I-restricted cytotoxic T cell responses induced by cross-presentation of viral antigens. J Immunol.

[55] Khan N, Bruton R, Taylor GS, Cobbold M, Jones TR, Rickinson AB (2005). Identification of cytomegalovirus-specific cytotoxic T lymphocytes in vitro is greatly enhanced by the use of recombinant virus lacking the US2 to US11 region or modified vaccinia virus Ankara expressing individual viral genes. J Virol.

[56] Manley TJ, Luy L, Jones T, Boeckh M, Mutimer H, Riddell SR (2004). Immune evasion proteins of human cytomegalovirus do not prevent a diverse CD8+ cytotoxic T-cell response in natural infection. Blood.

[57] Gilbert MJ, Riddell SR, Li CR, Greenberg PD (1993). Selective interference with class I major histocompatibility complex presentation of the major immediate-early protein following infection with human cytomegalovirus. J Virol.

[58] Scheller N, Furtwängler R, Sester U, Maier R, Breinig T, Meyerhans A (2008). Human cytomegalovirus protein pp 65: an efficient protein carrier system into human dendritic cells. Gene Ther.

[59] Delmas S, Martin L, Baron M, Nelson JA, Streblow DN, Davignon J-L (2005). Optimization of CD4+ T lymphocyte response to human cytomegalovirus nuclear IE1 protein through modifications of both size and cellular localization. J Immunol.

[60] Khan N, Cobbold M, Keenan R, Moss PAH (2002). Comparative analysis of CD8+ T cell responses against human cytomegalovirus proteins pp65 and immediate early 1 shows similarities in precursor frequency, oligoclonality, and phenotype. J Infect Dis.

[61] Forner G, Saldan A, Mengoli C, Gussetti N, Palù G, Abate D (2016). CMV-ELISPOT but not CMV-QuantiFERON assay is a novel biomarker to determine risk of congenital CMV infection in pregnant women. J Clin Microbiol.

[62] Saldan A, Forner G, Mengoli C, Tinto D, Fallico L, Peracchi M (2016). Comparison of cell-mediated immune assays CMV-ELISPOT and CMV-QuantiFERON in CMV seropositive and seronegative pregnant and non-pregnant women. J Clin Microbiol.

[63] Loeth N, Assing K, Madsen HO, Vindeløv L, Buus S, Stryhn A (2012). Humoral and cellular CMV responses in healthy donors; identification of a frequent population of CMV-specific, CD4+ T cells in seronegative donors. PLoS One.

[64] Lúcia M, Crespo E, Melilli E, Cruzado JM, Luque S, Llaudó I (2014). Preformed frequencies of cytomegalovirus (CMV)-specific memory T and B cells identify protected CMV-sensitized individuals among seronegative kidney transplant recipients. Clin Infect Dis Off Publ Infect Dis Soc Am.

[65] Sester M, Gärtner BC, Sester U, Girndt M, Mueller-Lantzsch N, Köhler H (2003). Is the cytomegalovirus serologic status always accurate? A comparative analysis of humoral and cellular immunity. Transplantation.

[66] Edson CM, Hosler BA, Respess RA, Waters DJ, Thorley-Lawson DA (1985). Cross-reactivity between herpes simplex virus glycoprotein B and a 63,000-dalton varicella-zoster virus envelope glycoprotein. J Virol.

[67] Vafai A, Wroblewska Z, Graf L (1990). Antigenic cross-reaction between a varicella-zoster virus nucleocapsid protein encoded by gene 40 and a herpes simplex virus nucleocapsid protein. Virus Res.

[68] Su LF, Kidd BA, Han A, Kotzin JJ, Davis MM (2013). Virus-specific CD4(+) memory-phenotype T cells are abundant in unexposed adults. Immunity.

[69] Kieper WC, Troy A, Burghardt JT, Ramsey C, Lee JY, Jiang H-Q (2005). Recent immune status determines the source of antigens that drive homeostatic T cell expansion. J Immunol.

[70] Yukl SA, Shergill AK, Girling V, Li Q, Killian M, Epling L (2015). Site-specific differences in T cell frequencies and phenotypes in the blood and gut of HIV-uninfected and ART-treated HIV+ adults. PLoS One.

[71] Dubois E, Ruschil C, Bischof F. Low frequencies of central memory CD4 T cells in progressive multifocal leukoencephalopathy. Neurol Neuroimmunol Neuroinflammation [Internet]. 2015. [cited Oct 24 2016];2. Available from: http://www.ncbi.nlm.nih.gov/pmc/articles/PMC4630684/. 10.1212/NXI.0000000000000177 PMC4630684 26568972

[72] Alanio C, Lemaitre F, Law HKW, Hasan M, Albert ML (2010). Enumeration of human antigen–specific naive CD8+ T cells reveals conserved precursor frequencies. Blood.

[73] Sprent J, Surh CD (2011). Normal T cell homeostasis: the conversion of naive cells into memory-phenotype cells. Nat Immunol.

[74] Hanley PJ, Melenhorst JJ, Nikiforow S, Scheinberg P, Blaney JW, Demmler-Harrison G (2015). CMV-specific T cells generated from naïve T cells recognize atypical epitopes and may be protective in vivo. Sci Transl Med.

[75] Hanley PJ, Shaffer DR, Cruz CRY, Ku S, Tzou B, Liu H (2011). Expansion of T cells targeting multiple antigens of cytomegalovirus, Epstein-Barr virus and adenovirus to provide broad antiviral specificity after stem cell transplantation. Cytotherapy.

[76] Brenchley JM, Douek DC, Ambrozak DR, Chatterji M, Betts MR, Davis LS (2002). Expansion of activated human naïve T-cells precedes effector function. Clin Exp Immunol.

[77] Maroof A, Beattie L, Kirby A, Coles M, Kaye PM (2009). Dendritic cells matured by inflammation induce CD86-dependent priming of naive CD8+ T cells in the absence of their cognate peptide antigen. J Immunol.

[78] Zippelius A, Pittet MJ, Batard P, Rufer N, de Smedt M, Guillaume P (2002). Thymic selection generates a large T cell pool recognizing a self-peptide in humans. J Exp Med.

[79] Whitmire JK, Eam B, Whitton JL (2008). Tentative T cells: memory cells are quick to respond, but slow to divide. PLoS Pathog.

[80] Veiga-Fernandes H, Walter U, Bourgeois C, McLean A, Rocha B (2000). Response of naïve and memory CD8+ T cells to antigen stimulation in vivo. Nat Immunol.

[81] Brehm MA, Mangada J, Markees TG, Pearson T, Daniels KA, Thornley TB (2007). Rapid quantification of naive alloreactive T cells by TNF-alpha production and correlation with allograft rejection in mice. Blood.

[82] Ben-Sasson SZ, Gerstel R, Hu-Li J, Paul WE (2001). Cell division is not a “clock” measuring acquisition of competence to produce IFN-gamma or IL-4. J Immunol.

