# Supplementary figures and images for: Validation of T-Track® CMV to assess the functionality of cytomegalovirus-reactive cell-mediated immunity in hemodialysis patients
Source: BMC Immunol. 2017 Mar 7;18:15. doi: 10.1186/s12865-017-0194-z (PMC5339958; doi:10.1186/s12865-017-0194-z)

**A**

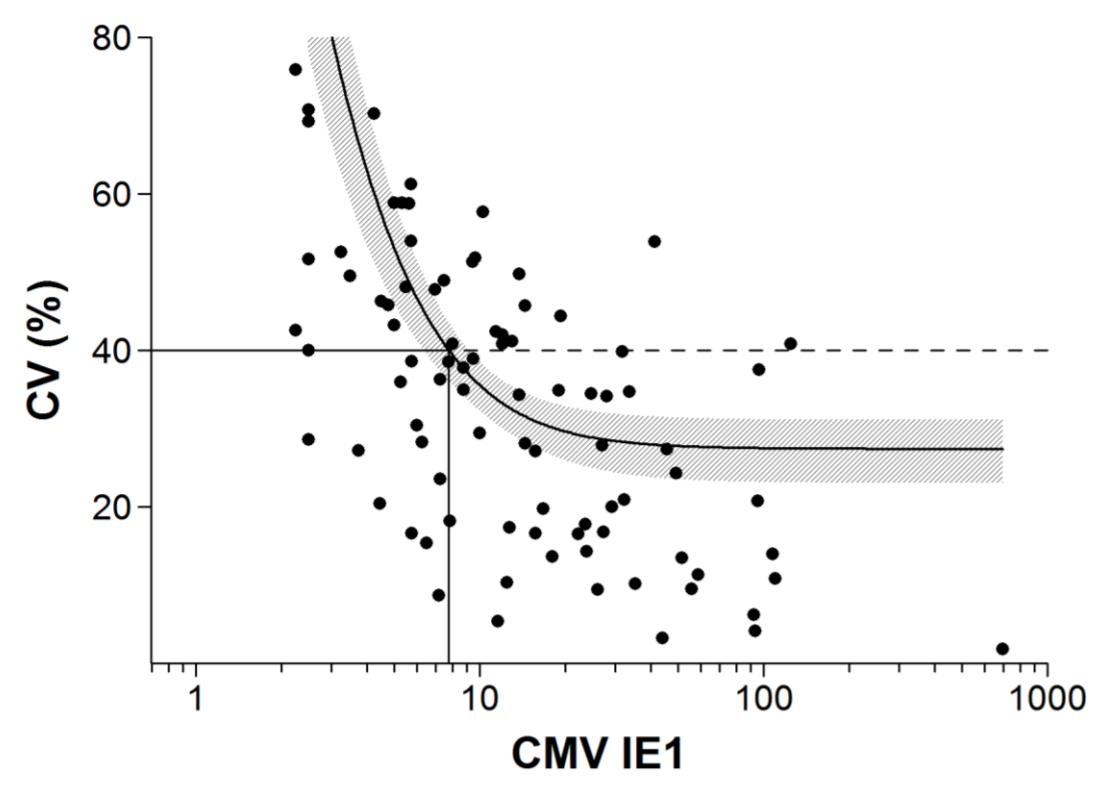

**B**

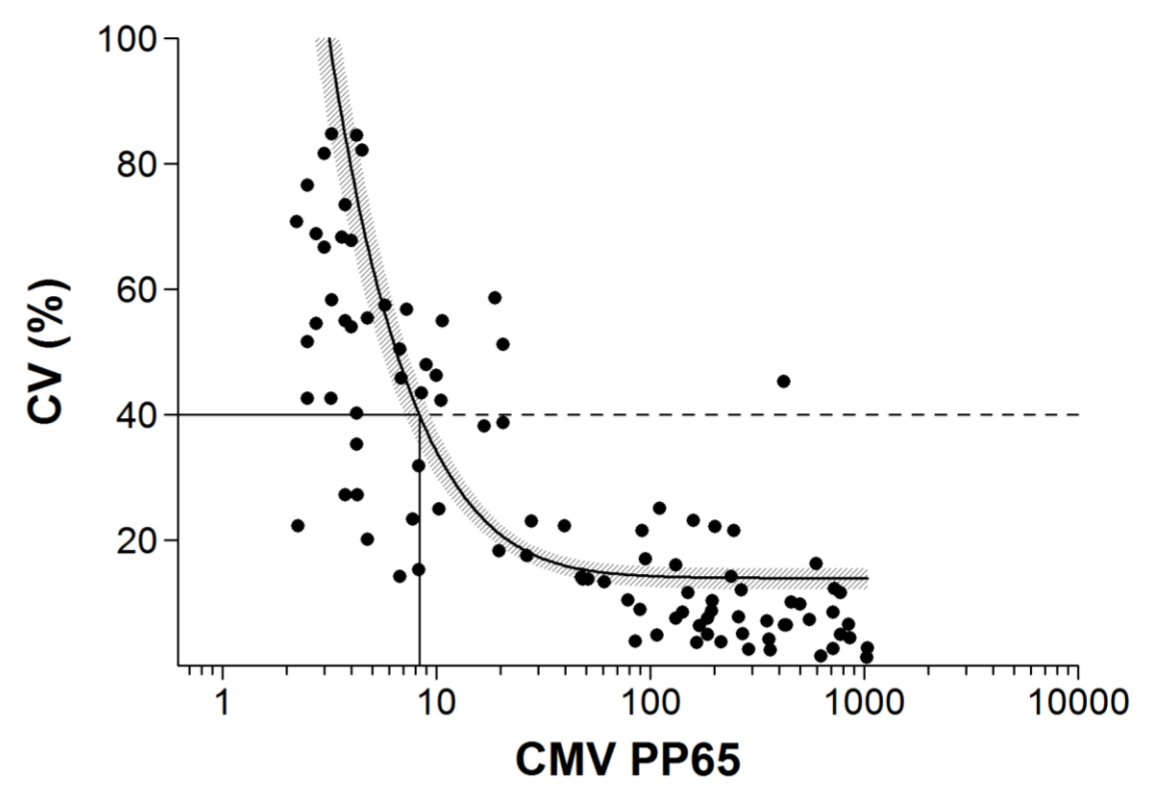

Additional File 1 (Banas et al.)

Supplement: Additional file 1: — Precision profiles of the specific response to IE-1 (A) and pp65 (B) in T-Track® CMV. A coefficient of variation (CV) no higher than 40% was used as a limit of acceptance of assay validity to determine the respective limit of quantitation (LoQ). LoQ values determined at CV = 40% for IE-1 (A) and pp65 (B) in the hemodialysis study (n = 124) using T-Track® CMV was 7.8 and 8.3 (SFC / 200,000 PBMC) respectively. Comparable LoQ values were obtained from T-Track® CMV assays performed on PBMC from 45 healthy donors [38]. Based on these analyses, a technical cut-off of 10 SFC / 200,000 PBMC was chosen. (PDF 334 kb) [file 12865_2017_194_MOESM1_ESM.pdf]
